# Anaemia at 36 weeks of pregnancy: Prevalence and determinants among antenatal women attending peri-urban facilities in a developing country, Ghana

**DOI:** 10.1371/journal.pgph.0003631

**Published:** 2024-09-05

**Authors:** Silas Adjei-Gyamfi, Abigail Asirifi, Wisdom Peprah, Delphina Aneley Abbey, Kwadzo Wisdom Hamenoo, Mary Sakina Zakaria, Osman Mohammed, Paul Armah Aryee

**Affiliations:** 1 Savelugu Municipal Hospital, Ghana Health Service, Savelugu, Northern Region, Ghana; 2 Department of Nutritional Sciences, School of Allied Health Sciences, University for Development Studies, Tamale, Northern Region, Ghana; 3 Department of Midwifery, Garden City University College, Kumasi, Ashanti Region, Ghana; 4 Faculty of Health and Allied Sciences, KAAF University College, Kasoa, Central Region, Ghana; 5 Begoro District Hospital, Ghana Health Service, Begoro, Eastern Region, Ghana; KEM Hospital Pune, INDIA

## Abstract

Anaemia as a critical health condition greatly upsurges the risk of pregnancy complications leading to preventable maternal mortalities and long-term morbidities. Therefore, identifying anaemia-associated factors is vital for planning relevant interventions in resource-constrained regions in Sahelian Africa. This study aimed to assess the prevalence and determinants of anaemia at 36 weeks of pregnancy among antenatal women in a peri-urban municipality of Ghana. A retrospective cross-sectional study was conducted among antenatal women from five different health facilities in Savelugu Municipality. Using antenatal register as the sampling frame, 422 participants were sampled. Data were collected via antenatal records review and a structured questionnaire. Using STATA, binary logistic regression was performed to identify significantly associated factors of anaemia at 36 weeks of pregnancy, considering a significance level of α = 0.05. Prevalence of anaemia at 36 weeks was 45.3%. Low socioeconomic status (AOR = 1.78; 95%CI:1.10–2.90; p = 0.020), pre-pregnancy body mass index ≥ 25 kg/m^2^ (overweight or obesity) (AOR = 1.62; 95%CI:1.01–2.58; p = 0.041), non-intake of sulphadoxine-pyrimethamine drugs (AOR = 2.22; 95%:1.40–3.51; p = 0.001), and malaria infection (AOR = 3.14; 95%CI:1.66–5.93; p<0.001) were associated with increased odds of anaemia at 36 weeks of pregnancy. Anaemia remains a burden in peri-urban Northern Ghana. Given the observed correlates of anaemia, interventions should be focused on strengthening malaria preventive measures, poverty alleviation, and peri-conception nutrition programs to avert adverse pregnancy outcomes.

## Introduction

Pregnancy-related anaemia is a critical pathophysiological state that is associated with serious health consequences [1, 2]. The World Health Organization (WHO) defines anaemia as a disorder where the quantity of erythrocytes or the oxygen‐carrying capacity of erythrocytes is inadequate to meet human physiological requirements [2, 3]. Clinically, anaemia (during pregnancy) is also regarded as low or reduced haemoglobin levels of less than 11.0 g/dl which is further categorized into mild (10.0–10.9g/dl), moderate (7.0–9.9g/dl), and severe (< 7.0g/dl) [2]. The measure of haemoglobin level remains the standard test for pregnant women during antenatal visits which is used to estimate and assess anaemia [2, 4]. WHO does not endorse the use of diverse haemoglobin cut-off ranges for diagnosing anaemia across all trimesters of pregnancy [2, 3], although it is established that during the second trimester, haemoglobin levels lessen by nearly 5.0–14.0 g/l [5]. As recommended by WHO, Ghana employs haemoglobin levels of <11.0 g/dl to determine anaemia among pregnant women across all three trimesters of pregnancy [6, 7].

Pregnancy increases susceptibility to anaemia due to stimulated physiological modifications [5]. The causes of anaemia are frequently triggered by antecedent factors including micronutrient (vitamin A, iron, and folic acid) deficiencies, low dietary diversity, underweight, and pre-existing diseases like malaria, and helminthiasis [8–12] leading to adverse production or abnormal loss of erythrocytes during pregnancy. Other significant predictors of anaemia in developing countries are rural residence, low socioeconomic status (SES), low educational status, marital status, low parity, late antenatal booking, low household income, non-patronization of family planning, and non-utilization of insecticide-treated nets [6, 13–15]

Pregnancy-related anaemia has largely been associated with adverse outcomes such as low “appearance, pulse, grimace, activity, and respiration” (APGAR) scores, intra-uterine growth retardation, mental impairment, miscarriages and abortions, preterm babies, small-for-date babies, low birthweight, macrosomia, perinatal, neonatal, and maternal mortalities [7, 15–22]. These devastating effects of anaemia during pregnancy could leave a remarkable toll on the national economy in most low and middle-income countries like Ghana. It is therefore imperative to regularly check haemoglobin levels in pregnancy and explore the factors associated with it for continuous updates of clinical and preventive services [6, 18].

Several interventions have been established to control and prevent gestational anaemia in most developing countries through community participation, educational system, and also during antenatal visits at primary to tertiary levels of health care. Some of these remarkable measures include girls’ iron folic acid tablet supplementation; infection prevention; micronutrient supplementation; mandatory haemoglobin testing at registration, 28 weeks, and 36 weeks of gestation; nutrition education and counseling; robust referral system; and malaria prevention via distribution of insecticide-treated bed nets and directly observed sulphadoxine-pyrimethamine (SP) intake [6, 7, 17, 18, 23, 24].

Despite the ongoing global interventions, anaemia still affects approximately 38.2% of total global pregnant mothers [2]. Southeastern Asia has the highest burden of gestational anaemia (49%) followed by African women with a prevalence of 46%. In Ghana, more than 40% of all pregnant women are anaemic [6, 25–28]. Notably, some cross-sectional and retrospective studies reported the prevalence of anaemia at the third trimester of pregnancy (36 gestational weeks) in China [14], Ghana [4], and Tanzania [22] to be 16.6%, 44.4%, and 47.4% respectively. A recent cross-sectional study conducted in the Savelugu municipality of Ghana revealed anaemia prevalence of 44.9%, 56.2%, and 44.4% at registration, 28 weeks, and 36 weeks of gestation respectively [7], however, this study did not assess the determining factors of this relatively higher pathophysiological state. Additionally, most Ghanaian studies on anaemia in pregnancy look at anaemia prevalence and its determinants at registration and sometimes at 28 weeks, without considering the third trimester of pregnancy [25–28]. We could not identify studies in Northern Region of Ghana (including Savelugu municipality) assessing haemoglobin levels and anaemia status among pregnant women at 36 weeks and its determinants. The 36 weeks of pregnancy is a crucial time for positive pregnancy outcomes as women approach their time of childbirth. Adequate knowledge on anaemia and its determinants in the third trimester of pregnancy especially at 36 weeks could be key in the antenatal care and childbirth process. Hence, this study assessed anaemia at 36 weeks of pregnancy and its associated sociodemographic, antenatal, and obstetric factors in a peri-urban municipality of Northern Ghana.

## Materials and methods

### Study setting

This study was conducted at health facilities providing antenatal care in the Savelugu Municipality of Northern Ghana. Savelugu Municipality is the nearest district to Tamale Metropolis in the Northern Region. The municipal has four sub-municipals with 28 health facilities including one hospital, two private clinics, four health centres, and 21 operational community-based health planning services (CHPS) zones providing services to a population of 129,283 of which 51.5% (66,581) are females. In 2019, the total women in their fertility age were 31,028 (24.0%) with a projected pregnancy rate of about 5,172 (4.0%) [16]. Out of the 28 health facilities, only five provided daily antenatal services with nationally recommended haemoglobin testing in 2019. In the same year, haemoglobin levels checked at 36 weeks of pregnancy was nearly universal (98%) [29]. Antenatal coverage was 94% in 2019 and nearly half of the women made at least eight antenatal visits before childbirth [7].

### Study design and population

The study employed a cross-sectional design and targeted pregnant women at 36 weeks of gestation attending antenatal services at five major public health facilities in Savelugu municipality. Women having twin gestation or with documented and/or reported genetic haematological problems or had experienced a severe form of bleeding during pregnancy were ineligible for the study. These women with the above conditions could have pathophysiological effects on erythrocytes production that are used to determine haemoglobin levels for the assessment of anaemia [2, 3, 5].

### Sample size and sampling

Using Yamane’s formula [30], the sample size (n) was initially determined as 383 with the following indicators; targeted antenatal registrants (N) of 7627 in 2019, and margin of error (e) of 5% at 95% confidence interval. Hence, $n=\frac{N}{1+Ne^{2}}=\frac{7,627}{1+(7,627\;x\;0.05\;x\;0.05)}$ ≈ 383.

After adding attrition of 10% [7] of the estimated study respondents (383), which was rounded off to 39, the final total sample size became 422.

During sampling, the five public health facilities providing daily antenatal services with nationally recommended haemoglobin testing procedures were included. The sample size per facility was determined by employing proportionate random sampling technique as shown in Table 1. In each facility, simple random sampling was then used to select the estimated participants by using the daily antenatal register as the sampling frame.

**Table 1:** Estimated facility sample size at the study setting.

	Savelugu municipal hospital	Savelugu health centre	Pong-Tamale health centre	Moglaa health centre	Diare health centre	Savelugu municipality (total)
**A:** Total 2019 antenatal visits at the health facilities	3,025	1,098	847	336	2,321	7,627
**B:** Total facility coverage (%) [= (A)/7627]	39.7%	14.4%	11.1%	4.4%	30.4%	100%
**C:** Total sample size for each facility [= (B) x 422]	167	61	47	19	128	422

### Data collection

Through a pretested questionnaire-based survey and antenatal records review, data were collected using trained research assistants (public health nurses) from 01 May to 31 December 2020. Sociodemographic characteristics, testing of haemoglobin levels by using venous blood, frequency of antenatal visits, and past medical and obstetric information of the sampled participants were extracted from antenatal record books. During data collection, anaemic pregnant women were educated and counseled on good nutrition and/or treatment protocols (including intake of prescribed drugs) by the public health nurses after referring them to see a medical officer. Data on socioeconomic characteristics (household assets), food security, maternal knowledge on anaemia, and additional information that were not recorded in the antenatal records were collected by using a structured questionnaire. A description of the key variables that were captured by the research assistants is exhibited in Table 2.

**Table 2:** Measurement and description of key variables.

Variable	Kind of variable	Explanation of variable	Categories used
Anaemia status	Binary	Haemoglobin levels less than 11g/dl	Anaemia or No anaemia
Severity of anaemia	Ordinal	Grouping anaemia status using WHO criteria	≥11g/dL = No anaemia; 10.0–10.9g/dL = Mild anaemia; 7.0–9.9g/dL = Moderate anaemia; and < 7g/dL = Severe anaemia.
Age group	Ordinal	The age category of mothers	16–25, 26–35, ≥36
Marital status	Nominal	Having customarily or legally bounded partner including being divorced or widowed	Single, Married, Widowed
Education	Ordinal	The highest educational level reached by mothers	No formal education, Primary, Junior high, Senior high, and Tertiary
Ethnicity	Nominal	Belonging to any traditional ethnic or tribal group	Dagomba, Gonja, Frafra, and Others (Asante, Ewe, Kusasi)
Religion	Nominal	The religious affiliation of mothers	Christianity, Islam, and Traditional
Occupation	Nominal	The working-class or economic sector operated by mothers	Unemployed, Informal, and Formal
Socioeconomic status (SES)	Ordinal	SES (as a proxy indicator of household wealth quintile) for principal component analysis of 16 selected household assets (water source, availability of electricity, cooking fuel type, toilet facility type, house roof material, maternal job status, house type, bicycle, mobile phone, television, car, radio, refrigerator, computer, motorcycle, and mattress/bed). This was further trichotomized into high, middle, and low SES [31]	Low, Middle, and High
Household food security	Ordinal	The degree of anxiety and uncertainty associated with food supply, food quality, food adequacy, and number of food intake by household members measured by the Food Insecurity Access Scale [4]	Food secure, Mild/Moderate food insecure, and Severe food insecure
Number of deliveries	Ordinal/ Binary	The number of deliveries by a respondent	0–1, and ≥2
Number of pregnancies	Ordinal/ Binary	The number of pregnancies by a respondent including the current pregnancy	0–1, and ≥2
Frequency of antenatal contacts	Ordinal/ Binary	The number of antenatal visits made by a woman during current pregnancy and categorized using 2016 WHO antenatal model	<8, and ≥8
Pre-pregnancy body mass index (BMI)	Ordinal	Defined as first-trimester body weight (kg) (thus, proxy pre-pregnancy weight since foetal weight gain in the first trimester is low) [32] divided by height (m^2^) and classified using WHO criteria.	<18.5kg/m^2^ = Underweight; 18.5–24.9kg/m^2^ = Normal; and ≥25kg/m^2^ = Overweight/obese
Sulphadoxine-pyrimethamine (SP) intake	Ordinal	The ingestion of SP since the respondent became pregnant	0, 1–3, and >3
Tetanus-diphtheria (TD) immunization	Binary	Determine whether the respondent has ever received TD immunization since pregnancy period	Yes or No
Insecticide-treated bed nets (ITNs) use	Binary	Determine whether the respondent sleeps under ITNs	Yes or No
Family planning (FP) use	Binary	The use of FP method before the current pregnancy	Yes or No
Iron folic acid (IFA) supplementation	Binary	The supply of IFA to a pregnant woman since she started antenatal services	Yes or No
Malaria infection	Binary	Episode of malaria infection throughout the current pregnancy	Yes or No
Previous history of anaemia	Binary	Women who had anaemia within a year before the most recent pregnancy.	Yes or No
Type of antenatal provider	Binary	The current care level of facility the respondent receives antenatal services	Hospital or Health centre
Knowledge on anaemia	Binary	Estimation of composited knowledge scores using a median cut-off point of 24 knowledge-related items before categorizing into adequate and inadequate knowledge level [7, 33]	Adequate or Inadequate

During the recruitment process, the research assistants first visited the selected health facilities to identify the eligible participants before sampling them for the study. The midwives at the health facilities aided the research assistants in the final contact and selection of the participants since the midwives were already aware of the visiting schedules of the pregnant women. The data were first recorded from the antenatal records before administering the questionnaire to the sampled participants through face-to-face interviews after the participants had consented to the study. The data collection per sampled participant lasted for 15–20 minutes.

### Data analysis

STATA version 17.0 (Stata Corporation, Texas, USA) was used to perform all analyses at a significance of p < 0.05. The outcome variable was anaemia at 36 weeks of pregnancy and exposure variables included all maternal background characteristics (sociodemographic, SES, obstetric, and antenatal variables). While numerical variables including age, haemoglobin levels, and knowledge scores were summarized using means and standard deviations, categorical variables such as educational level, gravidity, ethnicity, and job status were performed using frequencies and percentages. Principal component analysis was employed to construct wealth quintile (index) based on information collected on household assets before the wealth quintile was trichotomized [31]. Maternal first-trimester body mass index (BMI) was used as a proxy for pre-pregnancy BMI since foetal weight gain in the first trimester is low [32]. Maternal knowledge score was summed for each participant whilst 10 sets of questions were used with some of the questions having multiple responses. A correct answer was given one point while an inappropriate answer did not obtain any point. By applying the median cut-point, an absolute composite knowledge score was estimated using 24 items and dichotomized as adequate and inadequate knowledge level, with a probable lowest score of zero and the highest score of 30 [7, 33].

Univariate analysis and Chi-square/Fisher’s exact test were used to assess the association between the various background characteristics of the mothers and their anaemia status. Based on previous studies, significant determining factors (predictor variables) with p < 0.05 which are plausible and relevant in explaining pregnancy-related anaemia were forwarded into the multivariate (binary) logistic regression model after controlling for multicollinearity. Multicollinearity concerns among the predictor variables were determined by a linear regression model, and the predictor variables with a variance of inflation of less than 5 were ushered into the logistic analyses. Binary logistic regression was then computed to identify independent correlates of anaemia and the results were presented as adjusted odd ratios (AORs) within 95% confidence interval (CI).

### Ethical declarations

Navrongo Health Research Centre Institutional Review Board granted ethical approval for this study (approval number: NHRCIRB373). Permission was obtained from the Northern Regional Health Directorate, Savelugu Municipal Health Directorate, and heads of all sampled health facilities. Written informed consent/assent was obtained from all study participants and/or legal representatives and also from parents/guardians of participants under 18 years of age.

## Results

### Haemoglobin levels and anaemia at 36 weeks of gestation

Haemoglobin levels were documented for all sampled pregnant women at 36 weeks of pregnancy. The mean haemoglobin level was 10.7±1.6 g/dl with a median value of 11.0 g/dl. The prevalence of anaemia at 36 weeks was 45.3% (95%CI: 40.4%– 50.1%). Additionally, 23.9%, 19.0%, and 2.4% of the pregnant women had mild, moderate, and severe anaemia respectively (Table 3).

**Table 3:** Assessment of haemoglobin levels at 36 weeks of pregnancy.

Variables	Frequency (n)	Percent (%)	95%CI
**Haemoglobin (Hb) levels**			
Hb < 11g/dl (Anaemia)	191	45.3	40.4–50.1
Hb ≥ 11g/dl (No anaemia)	231	54.7	49.9–59.6
*Mean±sd*: *10*.*7±1*.*6 g/dl*			
**Severity of anaemia**			
Severe	10	2.4	1.1–4.3
Moderate	80	19.0	15.3–23.0
Mild	101	23.9	19.9–28.3

### Distribution of background characteristics among study participants

All the 422 recruited pregnant women fully responded to the study (Table 4). The mean age of the participants was 27.6 years with a standard deviation of 6.0. Most of the respondents were within the middle reproductive age group of 26–35 years (80.6%) and were coming from adequately food-secured households (78.0%). The greater proportion of the women had married partners (92.2%), were affiliated with the Islamic religion (88.2%), belonged to the municipal’s largest ethnic group (80.5%), had no formal education (43.1%), and were from low socioeconomic homes (40.3%). Additionally, majority of the women visited the antenatal clinic less than eight times (80.2%), were multigravidae (73.7%), and had normal pre-pregnancy BMI (66.1%). While most of the women were free from malaria infection during pregnancy (86.0%), slightly above two-thirds of the women also possessed adequate knowledge on anaemia (70.6%).

**Table 4:** Sociodemographic, obstetric, and antenatal factors associated with anaemia at 36 weeks of gestation.

Variables	Frequency distribution		Logistic regression analyses			
	Total	Anaemia	Univariate analysis		Multivariate analysis	
	N	N (%)	COR (95%CI)	p-value	AOR (95%CI)	p-value
***Sociodemographic variables***						
**Age group (years)**						
16–25	38	21 (55.3)	0.63 (0.32–1.24)	0.182		
26–35	340	149 (43.8)	1			
36–46	44	21 (47.7)	0.85 (0.46–1.60)	0.642		
**Marital status**						
Single	30	16 (53.3)	0.70 (0.33–1.47)	0.349		
Married	389	173 (44.5)	1			
Widowed	3	2 (66.7)	0.40 (0.04–4.45)	0.456		
**Education level**						
No education	182	86 (47.3)	1.27 (0.71–2.26)	0.417		
Primary	58	25 (43.1)	1.50 (0.73–3.09)	0.268		
Junior high	62	33 (55.2)	1			
Senior high	76	29 (38.2)	1.84 (0.93–3.64)	0.078		
Tertiary	44	18 (40.9)	1.64 (0.75–3.59)	0.212		
**Ethnicity**						
Dagomba	340	166 (48.8)	1		1	
Gonja	22	6 (27.3)	2.54 (0.97–6.66)	0.057	1.89 (0.66–5.45)	0.236
Frafra	34	10 (29.4)	2.29 (1.06–4.93)	0.034^†^	1.96 (0.88–4.40)	0.102
Others (Asante, Ewe, Kusasi)	26	9 (34.6)	1.80 (0.78–4.15)	0.167	1.64 (0.66–4.09)	0.289
**Religion**						
Islam	372	173 (46.5)	1			
Christianity	49	17 (34.7)	1.64 (0.87–3.04)	0.121		
Traditional	1	1 (100)	----	----		
**Occupation**						
Unemployed	153	77 (50.3)	0.80 (0.53–1.20)	0.284		
Informal	228	102 (44.7)	1			
Formal	41	12 (29.3)	1.95 (0.95–4.03)	0.068		
**Socioeconomic status**						
High	168	60 (35.7)	1.27 (0.75–2.14)	0.376	1.23 (0.70–2.13)	0.471
Middle	84	40 (47.6)	1		1	
Low	170	91 (53.5)	2.07 (1.34–3.21)	0.001^†^	**1.78 (1.10–2.90)**	**0.020** ^**†**^
**Household food security level**						
Food secure	329	150 (45.6)	0.66 (0.39–1.20)	0.185		
Mild/moderate food insecure	63	23 (36.5)	1			
Severe food insecure	30	18 (60.0)	0.38 (0.16–0.94)	0.085		
***Obstetric and antenatal variables***						
**Number of pregnancies**						
0–1	111	52 (46.9)	0.89 (0.56–1.39)	0.594		
≥ 2–4	251	110 (43.8)	1			
≥ 5	60	29 (48.3)	0.84 (0.47–1.47)	0.528		
**Number of deliveries**						
0–1	120	55 (45.8)	0.93 (0.60–1.45)	0.758		
≥ 2–4	247	109 (44.1)	1			
≥ 5	55	27 (49.1)	0.82 (0.46–1.47)	0.504		
**Frequency of antenatal contacts**						
< 8	304	147 (48.4)	1		1	
≥ 8	118	44 (37.3)	0.64 (0.41–0.98)	0.041^†^	0.80 (0.50–1.30)	0.378
**Pre-pregnancy body mass index (BMI)**						
Underweight (BMI < 18.5 kg/m^2^)	13	7 (53.8)	0.84 (0.27–2.56)	0.758	0.84 (0.25–2.80)	0.783
Normal (BMI 18.5–24.9 kg/m^2^)	279	138 (49.5)	1		1	
Overweight/obese (BMI ≥ 25 kg/m^2^)	130	46 (35.4)	1.79 (1.16–2.75)	0.008^†^	**1.62 (1.01–2.58)**	**0.041** ^**†**^
**Sulphadoxine-pyrimethamine intake**						
None	25	17 (68.0)	2.13 (1.40–3.26)	<0.001^†^	**2.22 (1.40–3.51)**	**0.001** ^**†**^
1–3 doses	249	126 (50.6)	1		1	
> 3 doses	148	48 (32.4)	0.48 (0.20–1.16)	0.103	0.56 (0.22–1.42)	0.227
**Tetanus-diphtheria immunization**						
No	31	19 (61.3)	1			
Yes	391	172 (44.0)	2.01 (0.95–4.26)	0.067		
**Insecticide-treated bed nets use**						
No	152	74 (48.7)	1			
Yes	270	117 (43.3)	1.24 (0.83–1.85)	0.289		
**Family planning use before current pregnancy**						
No	347	165 (47.6)	1		1	
Yes	75	26 (34.7)	1.71 (1.02–2.87)	0.044^†^	1.57 (0.22–1.43)	0.122
**Iron folic acid supplementation**						
No	7	5 (71.4)	1			
Yes	415	186 (44.8)	3.08 (0.59–16.0)	0.182		
**Malaria infection during pregnancy**						
No	363	153 (42.2)	1		1	
Yes	59	38 (64.4)	2.48 (1.40–4.40)	0.002^†^	**3.14 (1.66–5.93)**	**<0.001** ^**†**^
**Previous history of anaemia**						
No	394	178 (45.2)	1			
Yes	28	13 (46.4)	0.95 (0.44–2.05)	0.898		
**Type of antenatal provider**						
Health centre	228	101 (44.3)	1			
Hospital	194	90 (46.4)	0.92 (0.63–1.34)	0.667		
**Ever received education on anaemia**						
No	14	9 (64.3)	1			
Yes	408	182 (44.6)	2.23 (0.74–6.79)	0.156		
**Maternal knowledge on anaemia**						
Inadequate	124	57 (46.0)	1			
Adequate	298	134 (45.0)	1.04 (0.68–1.59)	0.851		

^*†*^ *p-value < 0*.*05 Regression model (R*^*2*^:*0*.*396; p<0*.*001) COR*: *crude odds ratio AOR*: *adjusted odds ratio Bold AOR*, *95%CI*, *p-value*: *significant values for associated factors*

### Factors associated with anaemia

The contributions of background (sociodemographic, socioeconomic, antenatal, and obstetric) variables as determinants of anaemia at 36 weeks of pregnancy were evaluated by binary logistic analyses (Table 4). Out of the seven background variables that showed univariate and/or bivariate associations with anaemia status, four variables were significant at the multivariate level after no multicollinearity issue was registered.

Pregnant women from poor households (low maternal SES) were 78% more likely to be anaemic compared to women from averagely-rich households (middle maternal SES) (AOR = 1.78; 95%CI:1.10–2.90; p = 0.020). Overweight or obese (thus, BMI ≥ 25 kg/m^2^) women were statistically (62%) more likely to be anaemic compared to those having normal BMI (AOR = 1.62; 95%CI:1.01–2.58; p = 0.041). Women who did not ingest SP drugs during the period of pregnancy were twice more likely to be anaemic compared to women who took about 1 to 3 doses of the SP drugs (AOR = 2.22; 95%:1.40–3.51; p = 0.001). Women who had malaria infection during pregnancy had three-fold increased odds of anaemia compared to those who were not infected (AOR = 3.14; 95%CI:1.66–5.93; p<0.001).

## Discussion

Anaemia in pregnancy may harm the national economy in numerous developing countries [2]. Additionally, the solicitous prevention and annulment of adverse pregnancy outcomes could be achieved through thorough identification of the contributing factors of pregnancy-related anaemia in most resource-constrained settings like Ghana. As a result, the study assessed the prevalence of anaemia and its correlates at 36 weeks of pregnancy during antenatal visits in the Savelugu municipality of Northern Ghana. This study determined an anaemia rate of 45.3% at 36 weeks of pregnancy, which is lesser than the rate in Blue Nile State, Sudan [34] and Jharkhand, India [35] of 64.7% and 86.0% respectively. Higher late antenatal registration (commonly in the second and third trimesters of pregnancy) is found in Sudan and India [34, 35] which increases the risk of gestational anaemia [2, 36] as compared to this study. There are no national estimates in Ghana to compare this study’s rate to, nonetheless, the anaemia rate in this study is slightly greater than some Ghanaian published estimates for Wa municipality (Upper West Region) [4] of 44.8%, and lesser than that of the Tamale Metropolis (36), Tatale-Sanguli/Zabzugu district (Northern Region) [37], and Bolgatanga Municipality (Upper East Region) [8] of 62.6%, 72.1%, and 81.5% respectively. The monthly supplementation of iron-folic acid tablets at antenatal clinics in Ghana and the regular intake by pregnant women over the past years could contribute to the reduced anaemia prevalence in our study as compared to that of the reported districts in Northern Region and Bolgatanga Municipality [4, 8]. Notwithstanding, cultural and contextual diversities among Ghanaian districts/municipalities are also more likely to be responsible for these observed differences in anaemia. The free maternal healthcare policy in Ghana is supposed to provide universal access to antenatal health services especially on haemoglobin testing, however, there are quality variations in haemoglobin testing across the regions and/or municipalities [38, 39] that could be accountable for these anaemia differences in the country. As pregnancy-related anaemia is still one of the leading causes of maternal deaths [40], it is of significant concern in Ghana [24]. We, therefore, propose addressing geographic-specific contributing factors including low maternal SES, overweight mothers, malaria infection, and non-intake of SP drugs to control and prevent anaemia during pregnancy.

Low maternal SES (thus, poor maternal household) was significantly associated with anaemia. Similar findings have been reported in other territories around the globe, notably in China [14], Ethiopia [9], and Nigeria [13]. The finding is also unparallel to that of Abaane and colleagues [4] who reported that pregnant women from rich households are associated with anaemia. Socioeconomic status as a social determinant of health influences food purchasing, food preferences, and dietary decisions among other social practices [41]. These social practices could range from appropriate food norms (diversified diets) to hygiene practices that can have an affirmative impact on anaemia prevention. As a result, women from low-SES households are mostly affected by malnutrition through their inability to afford foods rich in micronutrients and protein leading to the development of anaemia. Of the 93 women from food-insecure homes in our study, majority of them (n = 54; 58.1%) were from low SES households as few women were from high SES homes (p < 0.001 in Chi-square test). Hence, low SES homes are mostly food insecure which predisposes these homes to inappropriate diets [41] risking pregnant women to low haemoglobin formation [2]. SES is an essential driver that could be improved through designing and implementing interventions like home-based farming (plants and livestock) at the community level, especially in peri-urban settings. These interventions may not only increase economic flexibility for patronizing diversified diets but may also promote animal-source foods that are rich bioavailable sources of protein, iron, folate, and other micronutrients necessary for haemoglobin production.

The present study revealed that 30.8% (95%CI: 26.4%– 35.5%) of the women were overweight (obese) in the first trimester of pregnancy and had 1.62 times higher risk of anaemia. Several studies from different countries including Australia and Sudan reported the impact of overweight or obesity on anaemia in pregnancy [42, 43]. This finding is inconsistent with some retrospective studies in other settings [9, 14, 28]. Being overweight or obese increases anaemia through inflammatory response, as adiposity has been significantly linked to inflammation with potentially elevated C-reactive proteins [42, 44]. There is also an increase in hepcidin levels in the liver thereby inhibiting the absorption of iron and causing reduced haemoglobin concentration in the body [42]. As BMI increases with increasing muscular mass, being overweight or obese could also increase anaemia incidence due to inappropriate nutrition [44].

Consistent with previous studies [34, 35], the present study showed that anaemic mothers were infected with malaria during pregnancy while Nonterah and colleagues [28] documented no association between anaemia in pregnancy and malaria infection. Ghana is one of the malaria-prone regions of sub-Saharan Africa where *Plasmodium falciparum* is the main causative species [36], despite numerous interventions implemented to forestall this infection. The prevalence of gestational malaria infection in our study was estimated at 14.0% (95%CI:10.8%– 17.7%) which is higher than that of Volta Ghana (11.0%) [45]. This difference could be explained as a result of poor community adherence to malaria preventive measures in Northern Ghana as compared to Volta Ghana [6]. Anaemia as the most prominent haematological manifestation of malaria infection [35] is pathologized in malaria-infected pregnant women through haemolytic effects and increased erythrocytes’ clearance leading to decreased production of erythrocytes in the bone marrow [46, 47]. Depending on the severity, malaria can affect the placenta and foetus during pregnancy. It also serves as the principal cause of anaemia-associated morbidities and mortalities as well as adverse pregnancy outcomes like low birthweight, preterm births, and postpartum haemorrhage [47].

Through the intermittent preventive treatment of malaria in pregnancy (IPTp) program with directly observed ingestion of SP drug, 94.1% (95%CI: 91.4%– 96.1%) of the pregnant women in this study were enrolled. Notwithstanding, the study indicated that non-intake of SP drugs during pregnancy increased the risk of anaemia. This finding corroborates with some Ghanaian cross-sectional studies [36, 48] but is incongruent with a similar study in Burkina Faso [49]. Our study adds to the fact that there must be intensified education on IPTp-SP program in addition to other preventive measures for anaemia control in peri-urban Ghana [36], although this program is clinically contraindicated in women with positive glucose-6-phosphate dehydrogenase (G6PD) enzyme deficiency [48]. Not being on the IPTp-SP program predisposes pregnant women to a higher risk of malaria infection by causing increased parasitaemia in maternal blood which subsequently leads to the development of malaria-associated anaemia in pregnancy [45].

### Strengths and limitations of the study

The study analyzed anaemia at 36 weeks of pregnancy which is a critical point of assessment for pregnancy outcomes [8]. It allowed for an exploration of anaemia among pregnant women in planning for childbirth. The study was conducted at all five public health facilities geographically dispersed in the four sub-municipals which gives a fair representation of the participants in the municipality. Additionally, triangulation of data was ensured as haemoglobin information were collected through antenatal case review from two different source documents which could concurrently reduce data inconsistencies and recall bias. However, this study faced some limitations. Different haemoglobin testing machines with quality variations could be used in the sampled health facilities. This could influence haemoglobin values due to instrumentation errors. Another weakness of the study is measurement and documentation problems. This may occur as a result of the use of some essential secondary variables like first-trimester BMI, gestational age, and haemoglobin levels among others. Data on other factors like dietary intake (nutritional deficiencies) and sanitation characteristics were not investigated or collected which could contribute to anaemia and affect the study results.

## Conclusion

Anaemia remains a burden in the municipality as low maternal SES, being overweight or obese, non-enrollment in the IPTp-SP program, and malaria infection influenced the risk of anaemia at 36 weeks of pregnancy. Adherence to malaria preventive measures and enhanced enrollment in IPTp-SP during pregnancy should be tailored in the municipality through effective behaviour change communication. In addition, a strengthened poverty alleviation program via pro-poor policies is recommended to improve the socioeconomic status of women with subsequent enhancement of maternal nutrition and prevention of anaemia. Women should be empowered to engage in home-based gardening (especially vegetables and livestock farming) to increase accessibility to diversified diets for the prevention of overweight (obesity) before and during pregnancy. Also, weight control activities via daily exercise and health talks should be encouraged. We recommend that a regional and/or national cohort study should be conducted to include other contributing factors like diet and sanitation to adequately comprehend anaemia variations and their correlates at 36 weeks as well as other trimesters of pregnancy.

## Supporting information

S1 Data Datasets collected, generated, and/or analyzed for the present study. (DTA)
